# Deleterious Effects of Neonicotinoid Pesticides on Drosophila melanogaster Immune Pathways

**DOI:** 10.1128/mBio.01395-19

**Published:** 2019-10-01

**Authors:** John A. Chmiel, Brendan A. Daisley, Jeremy P. Burton, Gregor Reid

**Affiliations:** a Department of Microbiology and Immunology, The University of Western Ontario, London, Ontario, Canada; b Centre for Human Microbiome and Probiotic Research, Lawson Health Research Institute, London, Ontario, Canada; c Department of Surgery, The University of Western Ontario and St. Joseph’s Health Care, London, Ontario, Canada; Pacific Northwest National Laboratory

**Keywords:** *Drosophila*, *Lactobacillus*, dual oxidase, honey bee, host-microbe interactions, immune deficiency pathway, microbiota, neonicotinoid, pesticides, probiotics, reactive oxygen species, toxins

## Abstract

Neonicotinoid insecticides are common agrochemicals that are used to kill pest insects and improve crop yield. However, sublethal exposure can exert unintentional toxicity to honey bees and other beneficial pollinators by dysregulating innate immunity. Generation of hydrogen peroxide (H_2_O_2_) by the dual oxidase (Duox) pathway is a critical component of the innate immune response, which functions to impede infection and maintain homeostatic regulation of the gut microbiota. Despite the importance of this pathway in gut immunity, the consequences of neonicotinoid exposure on Duox signaling have yet to be studied. Here, we use a Drosophila melanogaster model to investigate the hypothesis that imidacloprid (a common neonicotinoid) can affect the Duox pathway. The results demonstrated that exposure to sublethal imidacloprid reduced H_2_O_2_ production by inhibiting transcription of the *Duox* gene. Furthermore, the reduction in *Duox* expression was found to be a result of imidacloprid interacting with the midgut portion of the immune deficiency pathway. This impairment led to a loss of microbial regulation, as exemplified by a compositional shift and increased total abundance of *Lactobacillus* and *Acetobacter* spp. (dominant microbiota members) found in the gut. In addition, we demonstrated that certain probiotic lactobacilli could ameliorate Duox pathway impairment caused by imidacloprid, but this effect was not directly dependent on the Duox pathway itself. This study is the first to demonstrate the deleterious effects that neonicotinoids can have on Duox-mediated generation of H_2_O_2_ and highlights a novel coordination between two important innate immune pathways present in insects.

## Abstract (precis)

Sublethal exposure to certain pesticides (e.g., neonicotinoid insecticides) is suspected to contribute to honey bee (Apis mellifera) population decline in North America. Neonicotinoids are known to interfere with immune pathways in the gut of insects, but the underlying mechanisms remain elusive. We used a Drosophila melanogaster model to understand how imidacloprid (a common neonicotinoid) interferes with two innate immune pathways—Duox and Imd. We found that imidacloprid dysregulates these pathways to reduce hydrogen peroxide production, ultimately leading to a dysbiotic shift in the gut microbiota. Intriguingly, we found that presupplementation with probiotic bacteria could mitigate the harmful effects of imidacloprid. Thus, these observations uncover a novel mechanism of pesticide-induced immunosuppression that exploits the interconnectedness of two important insect immune pathways.

## INTRODUCTION

Neonicotinoid insecticides are a class of neuro-active agrochemicals used to control pest organisms. They are currently the most widely used (∼20% of the global market) insecticides in the world, owing largely to affordability, flexible application, and long-lasting systemic activity in plant tissue (1). Imidacloprid (IMI), with a half-life exceeding 1,000 days in some cases (2), is the most commonly used neonicotinoid and has been detected in 52% and 66% of all fruits and vegetables in the United States and China, respectively (3). Further supporting its ubiquity in the environment, IMI was recently found present in 51% of honey samples globally sourced through a citizen science project (4).

Despite their success as a pesticide, neonicotinoids pose a threat to honey bees and other beneficial pollinators and may contribute to declining pollinator populations (5, 6). Honey bees exposed to neonicotinoids have growth defects (7), motor deficiencies (8), and behavioral abnormalities (9, 10). Moreover, neonicotinoids at sublethal concentrations have been shown to cause immunosuppression and increased susceptibility to fungal and viral pathogens in honey bees (11–13). Therefore, by reducing immune function and increasing susceptibility to infection, exposure to low-dose pesticides is believed to pose a threat to beneficial pollinators.

The insect gut microbiota is simultaneously controlled by the immune deficiency (Imd) pathway and the dual oxidase (Duox) pathway (14–17). The Imd pathway is used to control Gram-negative bacteria through peptidoglycan recognition and subsequent Relish-mediated induction of expression of antimicrobial peptides (18, 19). The Duox pathway is divided into an expression pathway and an activation pathway. The expression pathway is mediated by p38 activation through the mitogen-activated protein (MAP) kinase pathway (20). Activated p38 causes phosphorylation of activating transcription factor 2 (ATF2), which is a transcription factor for the *Duox* gene. Duox pathway activation is induced by recognition of pathogen-secreted uracil and yeast (21, 22). This drives phospholipase C-β (PLC-β)-mediated calcium efflux, which triggers the subsequent conformational changes required in DUOX for H_2_O_2_ generation. In the presence of chloride, DUOX can also convert hydrogen peroxide (H_2_O_2_) to HOCl, a potent antimicrobial compound (23). Together, the Imd and Duox pathways control the insect gut microbiota in both honey bees (24, 25) and Drosophila melanogaster (15).

Honey bees are intrinsically difficult to work with under controlled laboratory settings because of their stringent requirement for queen pheromone replacement and social hierarchy. Drosophila melanogaster is a suitable organism to model the effects of pesticides on the innate immune system of bees as both insects possess homologous nicotinamide acetylcholine receptors (the primary target of neonicotinoids) and share highly conserved innate immune systems (12, 26). A major advantage to this model is that the genome of D. melanogaster is well characterized and easily manipulated. This allows for generation of pathway mutants, which aids in the understanding of how factors, like pesticides, influence immune functionality of insects. Moreover, D. melanogaster possesses a simple microbiota that is dominated by culturable bacteria, low in diversity, and can be easily monitored via either culture-based CFU enumeration or molecular methods like quantitative PCR (qPCR)-based quantification and 16S rRNA gene sequencing to determine composition (27).

It has been shown that loss-of-function mutations in the Duox or Imd pathways cause increased microbial load and reduced longevity (15). Interestingly, oral supplementation with certain probiotic *Lactobacillus* spp. can modulate these pathways to increase activation even in times of immunosuppression (28, 29). We have previously demonstrated that supplementation with Lactobacillus plantarum Lp39 could mitigate IMI-induced susceptibility to septic infection with Serratia marcescens, a Gram-negative bacterial pathogen (29). Nevertheless, the relationship between the Duox pathway and the insect microbiota is still poorly understood, and the effect of neonicotinoids on the Duox pathway and the microbiota is inadequately characterized. Here, we utilize D. melanogaster (with a simplified microbiota largely dominated by Gram-positive *Lactobacillus* spp. and Gram-negative *Acetobacter* spp.) as a tractable and high-throughput model to investigate the relationship between the Duox pathway, regulation of the insect microbiota, and the effect of sublethal imidacloprid exposure. Thus, we hypothesized that sublethal IMI exposure will alter Duox pathway signaling and thereby affect microbicidal H_2_O_2_ production in D. melanogaster.

## RESULTS

### Imidacloprid exposure causes loss of microbial regulation in D. melanogaster.

**FIG 1:**
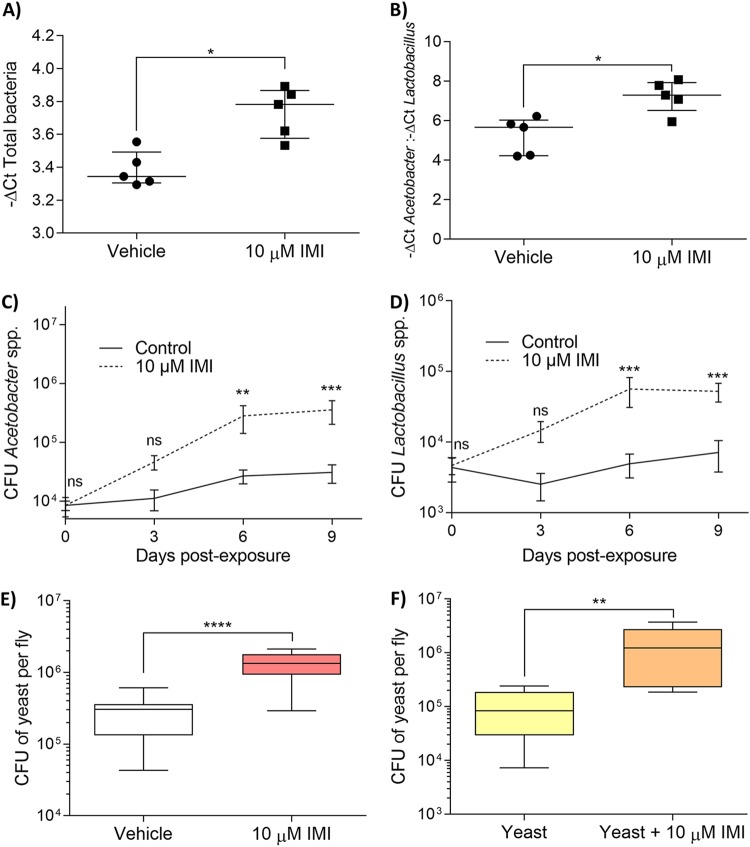
IMI exposure causes loss of microbial regulation in D. melanogaster. Three- to 5-day-old WT Canton-S flies were transferred to food vials containing vehicle (DMSO) or IMI (10 μM) for 5 days. Flies were then surface sterilized, DNA was extracted, and bacteria were quantified using qPCR microbial quantification relative to *Dros_rt_1* (*Drosophila* actin gene). Data are displayed as mean −Δ*C_T_* of total bacteria (A) or mean ratio of −Δ*C_T_* of *Acetobacter* to −Δ*C_T_* of *Lactobacillus* (B). Results are from 5 biological replicates (each consisting of 5 flies). Error bars represent median with interquartile range (Mann-Whitney test). (C and D) WT Canton-S time course CFU enumeration over 9 days of dominant gut bacteria per fly. Flies were surface sterilized and plated on MAN agar for *Acetobacter* spp. (C) and MRS agar for *Lactobacillus* spp. (D). Data are displayed as mean CFU per fly ± standard deviation (SD) (two-way ANOVA) at each time point from 3 biological replicates (*n* = 18 per time point for each group). (E and F) Three- to 5-day-old WT Canton-S flies were transferred to food vials containing either vehicle (DMSO) or IMI (10 μM) (E) or 2% (wt/vol) dried yeast (Saccharomyces cerevisiae) or 2% (wt/vol) dried yeast with 10 μM IMI (F) for 5 days. Flies were then surface sterilized and plated on YPD with 100 μg/ml of rifampin. Data are displayed as mean yeast CFU per fly ± SD (unpaired, two-tailed *t* test) from 12 biological replicates (each consisting of 5 flies). In box plot diagrams, boxes represent the first and third quartile values, while black lines denote medians. Whiskers encompass maximum and minimum values. *, *P* < 0.05; **, *P* < 0.01; ***, *P* < 0.001; ****, *P* < 0.0001; ns, not significant.

Quantitative PCR was used to determine the change in bacterial load in response to IMI exposure. Wild-type (WT) Canton-S exposed to IMI showed significantly higher threshold cycle (−Δ*C_T_*) values compared to control flies, which corresponds to a higher bacterial load (Mann-Whitney test; U = 1.000, *P* < 0.05) (Fig. 1A). The IMI-exposed flies also demonstrated a significant increase in the ratio of *Acetobacter* spp. to *Lactobacillus* spp. compared to control flies (Mann-Whitney test; U = 1.000, *P* < 0.05) (Fig. 1B). Time course CFU enumeration showed that the CFU of *Acetobacter* spp. and *Lactobacillus* spp. began to increase as early as 3 days after IMI exposure (Fig. 1C and D). Significant increases in both *Acetobacter* spp. (two-way analysis of variance [ANOVA]; *P* < 0.001) (Fig. 1C) and *Lactobacillus* spp. (two-way ANOVA; *P* < 0.0001) (Fig. 1D) were observed at days 6 and 9 of IMI exposure.

Drosophila melanogaster exposed to IMI was shown to have significantly higher abundance of total endogenous yeast per fly compared with control exposed flies (unpaired, two-tailed *t* test; *t* = 5.836, df = 22, *P* < 0.0001) (Fig. 1E). When D. melanogaster was administered 2% (wt/vol) Saccharomyces cerevisiae along with vehicle or IMI treatment, flies exposed to both IMI and the 2% yeast supplement had significantly higher CFU of yeast per fly compared to D. melanogaster given only the 2% yeast supplement (unpaired, two-tailed *t* test; *t* = 3.661, df = 22, *P* < 0.01) (Fig. 1F).

### Imidacloprid exposure affects Duox-mediated H_2_O_2_ production in D. melanogaster.

**FIG 2:**
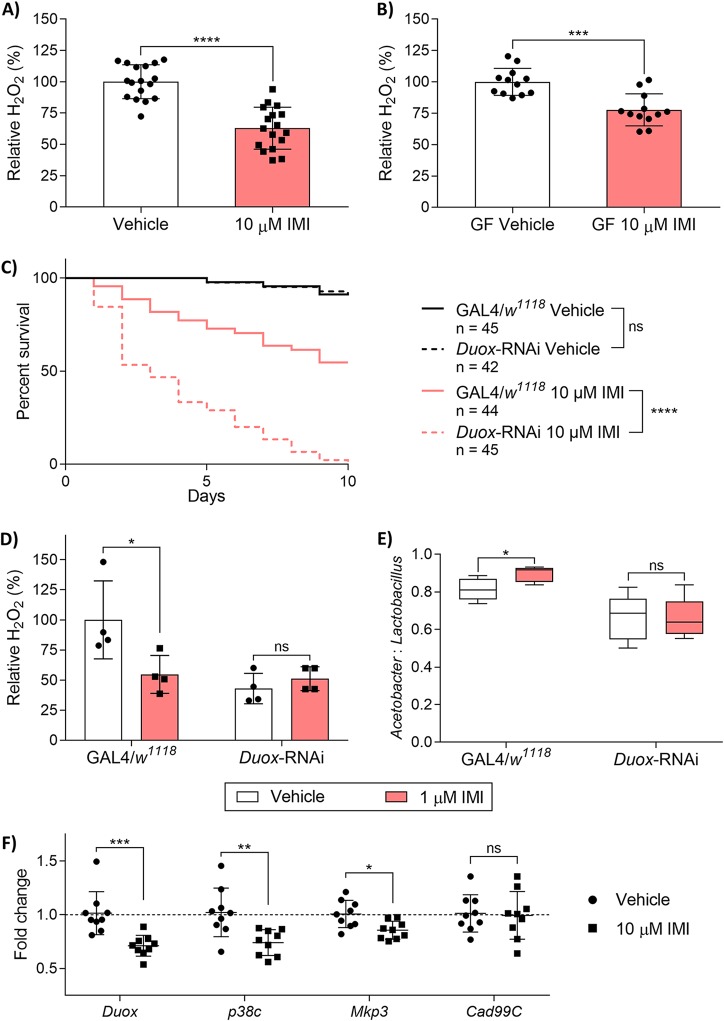
IMI exposure affects the Duox-mediated H_2_O_2_ production in D. melanogaster. Whole-body H_2_O_2_ concentrations of three female flies was measured using Amplex Red and normalized to total protein. (A and B) Three- to 5-day-old conventional WT Canton-S flies (A) and germfree (GF) WT Canton-S (B) were placed on vehicle (DMSO) or IMI (10 μM) for 5 days. Data are displayed as mean relative % of H_2_O_2_ ± SD (unpaired, two-tailed *t* test) from 17 biological replicates and 12 biological replicates (each consisting of 3 flies), respectively. (C) Survival curves for GAL4/*w^1118^* and *Duox*-RNAi on IMI (10 μM) or vehicle (DMSO) for 5 days. Data are displayed from at least 3 independent experiments (*n* = 15 to 25 for each group). Statistical analyses are shown from log-rank (Mantel-Cox) tests. (D and E) Three- to 5-day-old GAL4/*w^1118^* and *Duox*-RNAi flies were exposed to 1 μM IMI. (D) Whole-body H_2_O_2_ concentrations of three female flies were measured from flies exposed for 5 to 7 days. Data points represent mean relative % of H_2_O_2_ ± SD (Mann-Whitney tests) compared to GAL4/*w^1118^* of 4 biological replicates (each consisting of 3 flies). (E) CFU enumeration of the ratio of *Acetobacter* to *Lactobacillus* from flies exposed for 24 h. Flies were surface sterilized and plated on MAN agar for *Acetobacter* spp. and MRS agar for *Lactobacillus* spp. Data are displayed as mean *Acetobacter* CFU divided by total bacterial (*Acetobacter* + *Lactobacillus*) CFU ± SD (unpaired, two-tailed *t* tests) from 5 biological replicates, each consisting of 3 flies. (F) Gene expression of *Duox*, *p38c*, *Mkp3*, and *Cad99C* in WT Canton-S flies exposed to IMI (10 μM) or vehicle (DMSO) for 5 days. Data points are displayed as mean fold change (relative to *RpLP0*) of 5 pooled female flies in each group (*n* = 9). Error bars represent mean ± SD (Mann-Whitney test). In box plot diagrams, boxes represent first and third quartile values, while black lines denote medians. Whiskers encompass maximum and minimum values. *, *P* < 0.05; **, *P* < 0.01; ***, *P* < 0.001; ****, *P* < 0.0001; ns, not significant.

Since H_2_O_2_ is the primary metabolite produced downstream of the Duox pathway, its concentration was used to monitor pathway activity. Wild-type (WT) Canton-S flies exposed to sublethal (10 μM) IMI had significantly reduced whole-body H_2_O_2_ compared to vehicle-exposed flies (unpaired, two-tailed *t* test; *t* = 7.092, df = 32, *P* < 0.0001) (Fig. 2A). This was also observed in germfree (GF) flies, where IMI-exposed GF flies had significantly reduced whole-body H_2_O_2_ compared to vehicle-exposed GF flies (unpaired, two-tailed *t* test; *t* = 4.633, df = 22, *P* < 0.001) (Fig. 2B).

To test if the Duox pathway is necessary to resist IMI-induced toxicity, *Duox* RNA interference knockdown (*Duox*-RNAi) flies were exposed to IMI and assessed for survival. *Duox*-RNAi flies exposed to IMI demonstrated a significant reduction (log-rank [Mantel-Cox]; chi-square = 40.04, df = 1, *P* < 0.0001) in survival compared to control cross (GAL4/*w^1118^*) flies (Fig. 2C). There were no observable differences (Mann-Whitney test; U = 6, *P* = 0.6857) in whole-body H_2_O_2_ of *Duox*-RNAi flies exposed to either IMI or vehicle (Fig. 2D). Similar to our findings in WT flies, there was a significant decrease (Mann-Whitney test; U = 0, *P* < 0.05) in whole-body H_2_O_2_ of control cross (GAL4/*w^1118^*) flies exposed to IMI compared with vehicle-exposed control cross flies. In addition, there was no significant change in the ratio of *Acetobacter* spp. to *Lactobacillus* spp. of *Duox*-RNAi flies exposed to 1 μM IMI or vehicle (unpaired, two-tailed *t* test; *t* = 0.05109, df = 8, *P* = 0.9605) (Fig. 2E). Meanwhile, there was a significant increase in the ratio of *Acetobacter* spp. to *Lactobacillus* spp. for control cross (GAL4/*w^1118^*) flies exposed to 1 μM IMI compared with vehicle exposure (unpaired, two-tailed *t* test; *t* = 2.557, df = 8, *P* < 0.05).

As it appeared that the Duox pathway is involved in IMI toxicity, we looked at expression of Duox pathway-related genes in wild-type flies exposed to IMI (Fig. 2F). Canton-S flies exposed to sublethal IMI displayed a significant reduction in expression of *Duox* (Mann-Whitney test; U = 2, *P* < 0.001), *p38c* (Mann-Whitney test; U = 7, *P* < 0.01), and *MAP kinase phosphatase 3* (*Mkp3*) (Mann-Whitney test; U = 12, *P* < 0.05). These flies also displayed no change in *Cadherin 99C* (*Cad99C*) (Mann-Whitney test; U = 39.5, *P* = 0.9528) expression.

### Imidacloprid disrupts Duox expression via dysregulation of the Imd pathway.

**FIG 3:**
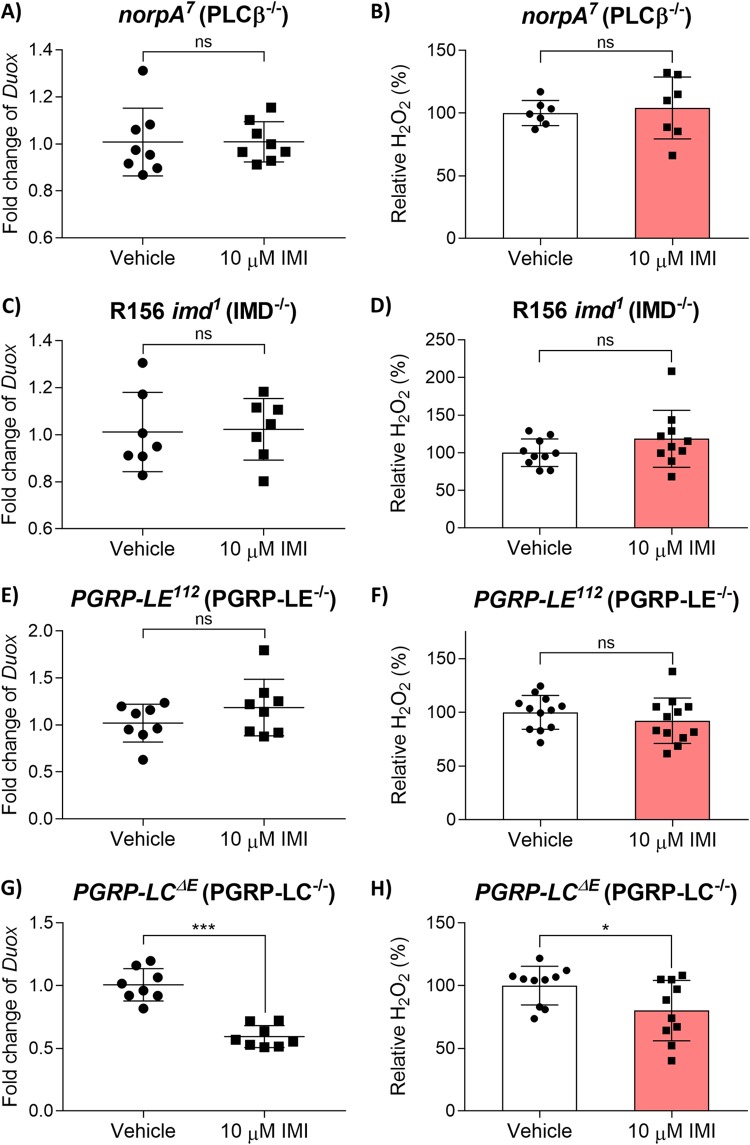
IMI impairs Duox pathway expression via the Imd pathway. (A and B) *norpA^7^* (PLC-β^−/−^) flies exposed to 10 μM IMI or vehicle (DMSO) for 5 days. (A) *Duox* gene expression data points are displayed as mean fold change (relative to *RpLP0*) of 8 biological replicates with 5 pooled female flies in each group. Error bars represent mean ± SD (Mann-Whitney test). (B) Whole-body H_2_O_2_ displayed as mean relative % of H_2_O_2_ ± SD (unpaired, two-tailed *t* test) from 7 biological replicates (each consisting of 3 flies). (C and D) R156 *imd^1^* (IMD^−/−^) flies exposed to 10 μM IMI or vehicle (DMSO) for 5 days. (C) *Duox* gene expression data points are displayed as mean fold change (relative to *RpLP0*) from 7 biological replicates with 5 pooled female flies in each group. Error bars represent mean ± SD (Mann-Whitney test). (D) Whole-body H_2_O_2_ displayed as mean relative % of H_2_O_2_ ± SD (unpaired, two-tailed *t* test) from 10 biological replicates (each consisting of 3 flies). (E and F) *PGRP-LE^112^* (PGRP-LE^−/−^) flies exposed to 10 μM IMI or vehicle (DMSO) for 5 days. (E) *Duox* gene expression data points are displayed as mean fold change (relative to *RpLP0*) from 8 biological replicates with 5 pooled female flies in each group. Error bars represent mean ± SD (Mann-Whitney test). (F) Whole-body H_2_O_2_ displayed as mean relative % of H_2_O_2_ ± SD (unpaired, two-tailed *t* test) from 12 biological replicates (each consisting of 3 flies). (G and H) *PGRP-LC*^Δ^*^E^* (PGRP-LC^−/−^) flies exposed to 10 μM IMI or vehicle (DMSO) for 5 days. (G) *Duox* gene expression data points are displayed as mean fold change (relative to *RpLP0*) from 8 biological replicates with 5 pooled female flies in each group. Error bars represent mean ± SD (Mann-Whitney test). (H) Whole-body H_2_O_2_ displayed as mean relative % of H_2_O_2_ ± SD (unpaired, two-tailed *t* test) from 10 biological replicates (each consisting of 3 flies). In box plot diagrams, boxes represent first and third quartile values, while black lines denote medians. Whiskers encompass maximum and minimum values. *, *P* < 0.05; **, *P* < 0.01; ***, *P* < 0.001; ****, *P* < 0.0001; ns, not significant.

To understand how IMI affects the expression of *Duox* and H_2_O_2_ generation, we exposed *norpA^7^* (PLC-β^−/−^) flies to 10 μM IMI and found that there no significant change (Mann-Whitney test; U = 27, *P* = 0.6454) in *Duox* expression (Fig. 3A). These flies also demonstrated no significant difference (unpaired, two-tailed *t* test; *t* = 0.4027, df = 12, *P* = 0.6943) in whole-body H_2_O_2_ (Fig. 3B).

Cross talk between the Imd and Duox pathways allows for coregulation of these two pathways. In particular, these two pathways converge on p38c, which is activated by the Imd pathway and regulates *Duox* transcription (30). Therefore, we assessed the potential of IMI to interfere with the cross talk between these pathways. We first exposed R156 *imd^1^* (IMD^−/−^) flies to IMI and found that there was no significant difference (Mann-Whitney test; U = 21, *P* = 0.7104) in *Duox* expression (Fig. 3C) or total-body H_2_O_2_ concentrations (unpaired, two-tailed *t* test; *t* = 1.388, df = 18, *P* = 0.1821) (Fig. 3D). Investigating upstream in the Imd pathway signaling cascade, we then exposed *PGRP-LE^112^* (PGRP-LE^−/−^) flies to 10 μM IMI or vehicle. We found no significant difference (Mann-Whitney test; U = 23, *P* = 0.3823) in *Duox* expression (Fig. 3E) and no significant difference in total-body H_2_O_2_ (unpaired, two-tailed *t* test; *t* = 1.015, df = 22, *P* = 0.3212) (Fig. 3F). We also exposed *PGRP-LC*^Δ^*^E^* (PGRP-LC^−/−^) flies to 10 μM IMI or vehicle. We found that there was a significant decrease (Mann-Whitney test; U = 0, *P* < 0.001) in *Duox* expression in IMI-exposed flies (Fig. 3G) and a significant reduction (unpaired, two-tailed *t* test; *t* = 2.199, df = 18, *P* < 0.05) in total-body H_2_O_2_ (Fig. 3H).

### Lactobacillus rhamnosus GR-1 supplementation mitigates imidacloprid-induced impairment of the Duox pathway and increases survival in D. melanogaster.

**FIG 4:**
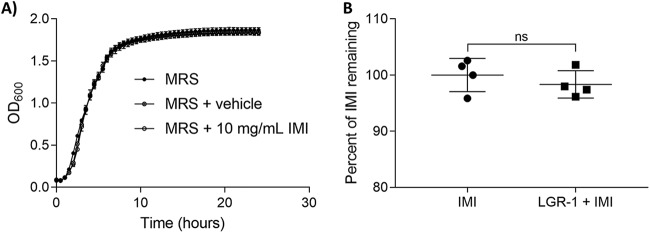
LGR-1 can survive with IMI but not remove it from solution. (A) Growth curve of LGR-1 in MRS and MRS supplemented with vehicle (DMSO) or 10 mg/ml IMI. Data points are depicted as mean ± SD from 3 biological replicates. (B) Percentage of IMI remaining in culture of LGR-1 grown in minimal media with yeast extract for 24 h. Data are displayed as mean % of IMI remaining ± SD from 4 biological replicates (Mann-Whitney test). *, *P* < 0.05; **, *P* < 0.01; ***, *P* < 0.001; ****, *P* < 0.0001; ns, not significant.

To test if human probiotic strain Lactobacillus rhamnosus GR-1 (LGR-1) would be a suitable supplement, we tested its ability to survive in culture with the addition of IMI. There were no apparent differences in the growth profile of LGR-1 grown in MRS supplemented with 100 μM IMI compared to growth in MRS alone (Fig. 4A). LGR-1 also demonstrated that it was not able to significantly reduce the concentration of IMI when grown *in vitro* (Mann-Whitney test; U = 6, *P* = 0.6857) (Fig. 4B).

**FIG 5:**
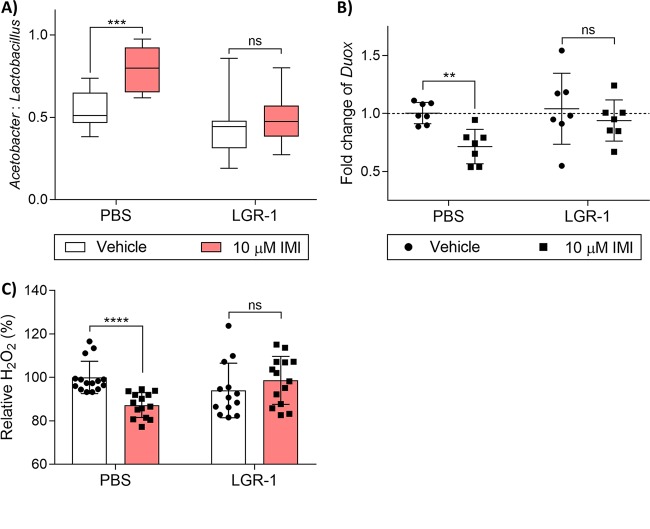
Probiotic supplementation improves survival of D. melanogaster exposed to IMI. (A) CFU enumeration of the ratio of *Acetobacter* to *Lactobacillus*. Flies were surface sterilized and plated on MAN agar for *Acetobacter* spp. and MRS agar for *Lactobacillus* spp. Data are displayed as mean *Acetobacter* CFU divided by total bacterial (*Acetobacter* + *Lactobacillus*) CFU ± SD (unpaired, two-tailed *t* tests) from 10 biological replicates (PBS vehicle), 8 biological replicates (PBS with 10 μM IMI), 9 biological replicates (LGR-1 vehicle), and 10 biological replicates (LGR-1 with 10 μM IMI), each consisting of 3 flies. (B) *Duox* gene expression displayed as mean fold change (relative to *RpLP0*) from 7 biological replicates with 5 pooled female flies in each group. Error bars represent mean ± SD (Mann-Whitney tests). (C) Whole-body H_2_O_2_ displayed as mean relative % of H_2_O_2_ ± SD (Mann-Whitney tests) compared to PBS vehicle of 15 biological replicates (PBS vehicle), 14 biological replicates (PBS with 10 μM IMI), 13 biological replicates (LGR-1 vehicle), and 14 biological replicates (LGR-1 with 10 μM IMI), each consisting of 3 flies. In box plot diagrams, boxes represent first and third quartile values while black lines denote medians. Whiskers encompass maximum and minimum values. *, *P* < 0.05; **, *P* < 0.01; ***, *P* < 0.001; ****, *P* < 0.0001; ns, not significant.

Wild-type (WT) Canton-S flies were presupplemented with LGR-1 or phosphate-buffered saline (PBS) for 48 h and then placed on vehicle (dimethyl sulfoxide [DMSO]) or 10 μM IMI to assess the ability of the bacterium to mitigate the sublethal effects of IMI. When LGR-1-supplemented WT Canton-S flies were exposed to a sublethal concentration (10 μM) of IMI, they showed no change in the gut ratio of *Acetobacter* spp. to *Lactobacillus* spp. (unpaired, two-tailed *t* test; *t* = 0.7744, df = 17, *P* = 0.4493) (Fig. 5A). The PBS-supplemented flies showed a significant increase in *Acetobacter* spp. (unpaired, two-tailed *t* test; *t* = 4.215, df = 16, *P* < 0.001) (Fig. 5A). Looking at the Duox pathway, LGR-1-supplemented flies fed sublethal IMI demonstrated no significant difference in *Duox* expression (Mann-Whitney test; U = 20, *P* = 0.5962) (Fig. 5B) and H_2_O_2_ (Mann-Whitney test; U = 68, *P* = 0.2800) (Fig. 5C) compared with LGR-1-supplemented vehicle-exposed flies. As seen with previous experiments, PBS-supplemented flies exposed to IMI showed reduced *Duox* expression (Mann-Whitney test; U = 2, *P* < 0.05) (Fig. 5B) and reduced H_2_O_2_ (Mann-Whitney test; U = 8, *P* < 0.0001) (Fig. 5C) compared to PBS-supplemented vehicle-treated flies.

## DISCUSSION

This study demonstrated that sublethal IMI exposure interferes with the Duox pathway in D. melanogaster. IMI-induced immunosuppression was observed by an increase in total bacteria and yeast, which has been associated with impaired Duox (22) and Imd (29) pathway function. There was a shift in the gut microbiota from a homeostatic balance of *Lactobacillus* spp. and *Acetobacter* spp. toward an *Acetobacter*-dominated gut microbiota upon exposure to IMI. However, this was not the case for *Duox-*RNAi flies exposed to IMI, indicating that the Duox pathway may be critical for mediating the gut-perturbing effects of IMI. *Acetobacter* colonization has been attributed to triacylglyceride reduction (31) and shortening of life span in D. melanogaster (32). Furthermore, *Acetobacter* spp. are known to accelerate larval development via increased insulin signaling (33), which has coined the idea that colonization with *Acetobacter* confers a “live fast, die young” lifestyle (32).

Hydrogen peroxide and other reactive oxygen species (ROSs) are essential molecules generated by the immune system to control gut homeostasis (34). We found that H_2_O_2_ was reduced in both GF and conventional WT Canton-S flies exposed to IMI, which suggests that IMI is directly interacting with the host to elicit Duox impairment and that this effect is not a result of an altered microbiota. Corroborating this, honey bee hemocytes exposed to imidacloprid show reduced H_2_O_2_ levels *in vitro* (35). Despite the potential regulatory interactions that occur between different microbial species, reduced H_2_O_2_ levels in the lumen of the intestinal tract are suspected to be the most likely candidate responsible for the observed shift in the gut microbiota. Interestingly, Duox pathway knockout flies have increased amounts of *Acetobacter* (16), further supporting the role of Duox in controlling Gram-negative spp. in the gut. Given that many lactobacilli are inherently resistant to ROSs (36), we propose that reduced H_2_O_2_ levels during IMI exposure would permit the growth of ROS-susceptible organisms (like *Acetobacter* spp.), and thereby reduce the relative abundance of *Lactobacillus* spp. via competitive exclusion.

Reactive oxygen species are a product of many metabolic processes in D. melanogaster; therefore, it is important to confirm that IMI is impairing Duox pathway production of ROS and not one of the other generators of ROS. We found that there was no significant difference between the H_2_O_2_ concentration of *Duox* RNAi flies exposed to IMI and vehicle, which suggests the Duox pathway is affected by IMI exposure. Corroborating our findings that show reduced *Duox* expression by IMI, it appears that the decrease in H_2_O_2_ observed in IMI-exposed WT Canton-S flies is a result of decreased *Duox* expression and is not mediated through direct impairment of the DUOX protein. Furthermore, activation-related components of the Duox pathway appear to be unaffected by IMI. In particular, *Cadherin 99C* (*Cad99C*) expression, which has been shown to be induced by uracil (an activator of the Duox pathway) (21), remained unchanged between vehicle- and IMI-exposed WT flies. In essence, it appears that Duox pathway functionality is intact, but expression is reduced, thus leading to reduced H_2_O_2_.

The Duox pathway is regulated by its own activation (22) and at the expression level by the Imd pathway (20). Since *Duox* expression was reduced, we first looked at how IMI affects Duox pathway signaling. We found that expression of *Mkp3* (a negative regulator of *Duox* expression) (20) and *p38c* (an activator of ATF2 transcription factor leading to *Duox* transcription) (30) was reduced in IMI-exposed flies. Moreover, there was no change in *Cad99C* (regulated by hedgehog signaling and associated with Duox pathway activation) (37). These results suggest that expression of *Duox* is not being inhibited by a negative regulator, nor by inadequate activation, but is impaired at the level of transcriptional activation of *Duox*. PLC-β knockout (*norpA^7^*) flies exposed to IMI showed no change in *Duox* expression or H_2_O_2_ concentration, likely because it functions downstream of Duox. Therefore, IMI is not directly acting on the Duox pathway to cause reduced *Duox* gene expression.

We investigated the Imd pathway because it can modulate *Duox* expression through peptidoglycan-dependent activation of p38 (20, 38). The R156 *imd^1^* (IMD^−/−^) flies exposed to IMI showed no change in *Duox* expression or H_2_O_2_ concentrations compared with vehicle-exposed flies. These flies lack a functional IMD protein; therefore, the absence of a change in *Duox* expression and H_2_O_2_ in IMI-exposed flies suggests that the Imd pathway is involved in mediating IMI-induced suppression of *Duox*. Imd pathway activation is achieved by peptidoglycan recognition receptors PGRP-LC and PGRP-LE. PGRP-LC mainly functions in the foregut, hindgut, and fat body as a surface receptor found on the impenetrable cuticle (39). PGRP-LE functions primarily in the midgut as an intracellular receptor, which binds molecules that cross the permeable peritrophic matrix (39, 40). PGRP-LC^−/−^ flies exposed to IMI showed a reduction in *Duox* expression and H_2_O_2_ levels, which suggests that IMI is not acting through this receptor to impair the Duox pathway. Rather, PGRP-LE^−/−^ flies exposed to IMI showed no change in *Duox* expression and no change in H_2_O_2_ concentration, which suggests that IMI may be acting through PGRP-LE to hinder the Duox pathway. Given the interconnectedness of the two pathways, this makes sense as both the Duox pathway and PGRP-LE function to control gut immunity (28, 40).

In brief, our data suggest that IMI might be interacting with the Imd pathway in the gut, which is influencing the Duox pathway by reducing *Duox* expression and H_2_O_2_ generation. These results are corroborated by studies that have shown that neonicotinoids interfere with NF-κB signaling and increase susceptibility to pathogen challenge in D. melanogaster and honey bees (12, 29, 41).

Supplementation with LGR-1 restored the balance in the gut microbiota and mitigated IMI-induced changes in the Duox pathway. Despite the ability of LGR-1 to inherently produce ROS (42), its effectiveness is likely attributed to its role in stimulating the host immune system. Gram-positive bacteria can be detected by PGRP-SD (43), which in turn can activate PGRP-LE and the subsequent Imd pathway (44). This activation of the Imd pathway can lead to p38-dependent Duox pathway expression (30), thereby alleviating the immune impairment induced by IMI. Moreover, LGR-1 is not able to metabolize or sequester IMI, thus promoting the notion of immune stimulation. Although it is difficult to directly extrapolate our findings to honey bees, similarities in immune responses to neonicotinoids (45) and bacterial probiotics (46) suggest that lactobacillus supplementation could bolster honey bee resistance to neonicotinoids.

In summary, this study shows that (i) exposure to IMI causes loss of microbial regulation by increasing Gram-negative bacteria and yeast, both regulated primarily by the Duox pathway, (ii) IMI exposure impairs *Duox* expression, leading to reduced antimicrobial H_2_O_2_, (iii) IMI-induced Duox pathway impairment might be acting through the Imd pathway in the midgut, and (iv) LGR-1 supplementation mitigates IMI-mediated Duox pathway impairments. Further work is merited on understanding the mechanism in which IMI interferes with the Imd pathway, investigating how lactobacilli mitigate IMI-induced suppression of Duox, and extending our findings to off-target species like honey bees.

## MATERIALS AND METHODS

### Chemicals.

Imidacloprid (catalog no. 37894) was obtained from Sigma-Aldrich. Stock solutions were prepared at 100 mg/ml in dimethyl sulfoxide (DMSO; Sigma-Aldrich) and stored at 4°C until usage.

### Drosophila melanogaster husbandry.

Wild-type (WT) Canton-S (stock no.1; RRID:BDSC_1), *w^1118^* (stock no. 3605; RRID:BDSC_3605), *daughterless* GAL4 (*da*-GAL4; stock no. 55850; RRID:BDSC_55850), *PGRP-LE^112^* (PGRP-LE^−/−^; stock no. 33055; RRID:BDSC_33055), and *PRGP-LC*^Δ^*^E^* (PGRP-LC^−/−^; stock no. 55713; RRID:BDSC_55713) flies were obtained from the Bloomington Drosophila Stock Center (NIH P40ODO18537) at Indiana University. The previously described UAS-*dDuox*-RNAi (*Duox*-RNAi) fly line (with approximately 50% reduction of *Duox*) (23) and R156 *imd^1^* (IMD^−/−^) fly line (47) were also used in this study. D. melanogaster flies were maintained using media with 1.5% (wt/vol) agar, 1.73% (wt/vol) yeast (Sigma-Aldrich catalog no. 51475), 7.3% (wt/vol) cornmeal, 7.6% (vol/vol) corn syrup, and 0.58% (vol/vol) propionic acid at 25°C with 12-h light/dark cycles. For experimental procedures, IMI media were supplemented with pesticide, and vehicle media were supplemented with dimethyl sulfoxide (DMSO) prior to agar solidification. All experiments were performed in wide polypropylene D. melanogaster vials (model no. GEN32-121 and GEN49-101; Diamed Lab Supplies, Inc., Mississauga, ON, Canada). Adult flies used for experiments were 3 to 5 days old unless otherwise stated. UAS × GAL4 crosses were performed by mating male *da*-GAL4 flies with virgin female UAS-*dDuox*-RNAi knockdown flies or virgin female *w^1118^* flies as a control. The GAL4 driver, *da*-GAL4, is an all-tissue driver, which has ubiquitous GAL4 expression. WT Canton-S flies were supplemented with 10 μM IMI, as previously determined to be sublethal (29). The sublethal dose of IMI for *Duox*-RNAi and GAL4/*w^1118^* flies was determined to be 1 μM (see Fig. S1 in the supplemental material).

10.1128/mBio.01395-19.1 FIG S1 Determination of sublethal IMI dose for *w^1118^* flies. Three- to 5-day-old *w^1118^* flies were exposed to vehicle (DMSO) or various concentrations of IMI to assess the sublethal dose. Data are displayed from at least 3 independent experiments (*n* = 25 to 30 for each group). Statistical analyses are shown from log-rank (Mantel-Cox) tests. *, *P* < 0.05; **, *P* < 0.01; ***, *P* < 0.001; ****, *P* < 0.0001; ns, not significant. Download FIG S1, TIF file, 0.1 MB. Copyright © 2019 Chmiel et al. 2019 Chmiel et al. This content is distributed under the terms of the Creative Commons Attribution 4.0 International license.

### Generation and rearing of germfree Drosophila melanogaster.

Germfree flies were prepared and reared on sterile media (48). Eggs were collected, rinsed with water to remove excess debris, and dechlorinated with 2.7% (vol/vol) sodium hypochlorite for 2 to 3 min, followed by two rinses with 70% ethanol. Finally, eggs were rinsed with sterile water for 10 min and placed on sterile media to grow. Germfree conditions were verified by homogenizing and plating D. melanogaster larvae on brain heart infusion (BHI), MRS, and mannitol (MAN) agar (3 g Bacto peptone no. 3, 5 g yeast extract, 25 g mannitol, 15 g agar, 1 liter H_2_O) and incubating them at 30°C for 2 days.

### DNA extraction for qPCR-based quantification of D. melanogaster gut bacteria.

Three- to 5-day-old Canton-S flies were placed on media containing 10 μM IMI or vehicle for 5 days. Five female flies were surface sterilized with 70% ethanol for 1 to 2 min and washed with sterile water. Flies were kept at –20°C until DNA extraction was performed. DNA was extracted using the method from Staubach et al. (49) with the Qiagen QIAmp DNA minikit (Qiagen catalog no. 51304). Briefly, flies were homogenized in 180 μl of ATL buffer containing 20 μl of proteinase K at 56°C for 30 min to soften the exoskeleton. Following this incubation, flies were homogenized by bead beating at 4,800 rpm with 0.1-mm (zirconia/silica; BioSpec catalog no. 11079101z), 0.5-mm (zirconia/silica; BioSpec catalog no. 11079105z), and 1-mm (glass) beads using a BioSpec 3110BX Mini Beadbeater 1 (Fisher Scientific catalog no. NC0251414) for 3 to 5 min with another incubation for 30 min at 56°C. Next, 200 μl of lysis buffer AL was added, and samples were incubated at 70°C for 30 min and then 95°C for 10 min. The rest of the extraction followed the manufacturer’s protocol. The quality of DNA was evaluated using a DeNovix DS-11 spectrophotometer and determined to have *A*_260/280_ and *A*_260/230_ absorbance ratios of between 1.7 to 1.9 and 1.7 to 2.2, respectively.

### Culture-based enumeration of D. melanogaster gut bacteria.

Three female flies were surface sterilized with 70% ethanol then homogenized with three 2-mm glass beads in 300 μl of PBS using a BioSpec 3110BX Mini Beadbeater 1 (Fisher Scientific catalog no. NC0251414). Homogenates were then serially diluted in PBS and plated on MRS and MAN agar. MRS plates were grown anaerobically at 30°C for 48 h, and MAN plates were grown aerobically at 30°C for 48 h. Subsequent CFU on MRS and MAN plates were counted and confirmed to be *Lactobacillus* spp. or *Acetobacter* spp., respectively, based on morphological characteristics and Gram stain analysis.

### D. melanogaster gut abundance of yeast.

Three- to 5-day-old Canton-S flies were exposed to vehicle (DMSO), 10 μM IMI, 2% (wt/vol) Saccharomyces cerevisiae (Fleischmann’s traditional active dry yeast) with vehicle, or 2% S. cerevisiae with 10 μM IMI on previously described media without the addition of propionic acid to allow the yeast to survive. Tubes consisted of 25 to 30 flies that were then kept under standard conditions for 5 days. Five female flies were surface sterilized and collected in 500 μl of PBS and then homogenized for 30 s at 4,800 rpm with three 2-mm glass beads. Homogenates were serially diluted and plated on YPD agar (10 g yeast extract, 20 g peptone, 20 g dextrose, 15 g agar, 1 liter double-distilled water [ddH_2_O]) with 100 μg/ml rifampin as previously described (22) and then incubated at 30°C for 24 to 48 h.

### Determination of H_2_O_2_-specific ROS in D. melanogaster.

Hydrogen peroxide was quantified using the Amplex Red hydrogen peroxide/peroxidase assay kit (Invitrogen catalog no. A22188) as previously demonstrated but with minor modifications (30). Three female adult D. melanogaster flies were collected and homogenized in 300 μl of PBS with three 2-mm glass beads beating for 10 s at 4,200 rpm. For Canton-S flies, heads were removed because of the intense red eye pigment. Samples were centrifuged at 12,000 × *g* for 3 min (room temperature), and 50 μl of supernatant was used for the assay following the manufacturer’s protocol with spectrophotometry quantification at 560 nm or excitation/emission of 535/595 nm using a BioTek Eon microplate reader or Eppendorf PlateReader AF2200, respectively. Hydrogen peroxide concentrations were normalized to total protein and plotted as relative H_2_O_2_ to the vehicle. Total protein was quantified using a bicinchoninic acid (BCA) protein assay kit (Invitrogen catalog no. 23227) following the manufacturer’s microplate protocol. Protein was measured from samples that were obtained from the H_2_O_2_ determination protocol and used to normalize H_2_O_2_ quantification. Samples were centrifuged at 12,000 × *g* for 3 min (room temperature), and 25 μl was used for quantification as per the manufacturer’s microplate protocol using a BioTek Eon microplate reader at 562 nm.

### Adult D. melanogaster survival assays.

Five- to 10-day old flies were used for all adult survival experiments as described previously (29) with modifications. Prior to the experimental start point, flies were gently anaesthetized with CO_2_ and transferred from standard rearing medium to an empty vial containing a 100-μl ddH_2_O-soaked Whatman filter disc (25 mm; Sigma-Aldrich) and starved for 120 min to normalize feeding frequency. For lethal exposure experiments, flies were briefly anesthetized with CO_2_ and transferred to vials with 5% sucrose agar (5% sucrose [wt/vol] and 1.5% agar [wt/vol]) containing 10 μM IMI or vehicle (DMSO). Any early deaths (<1 h) were assumed to be from the transfer process and removed from subsequent analyses. Survival was monitored daily at 24-h intervals from the experimental start point.

### RNA extraction and reverse transcription.

Five female adult D. melanogaster flies were homogenized in 550 μl of TRIzol reagent (Ambion catalog no. 15596018) using three 2-mm glass beads beating twice for 30 s at 4,800 rpm with a BioSpec 3110BX Mini Beadbeater 1 (Fisher Scientific catalog no. NC0251414). Tubes were centrifuged at 13,000 rpm for 10 min at 4°C to pellet debris. Supernatant was collected, and 0.2 volume of chloroform was added, followed by centrifugation at 13,000 rpm for 15 min at 4°C. The upper aqueous layer was collected, and 0.7 volume of isopropanol was added to precipitate the RNA, followed by centrifugation at 13,000 rpm for 15 min at 4°C. The RNA pellet was washed with 1 ml of 70% ethanol in diethyl pyrocarbonate-treated ddH_2_O and centrifuged at 13,000 rpm for 15 min at 4°C. Following removal of supernatant, the RNA was air dried and then resuspended in 30 μl of nuclease-free water. The quality of RNA was evaluated using a DeNovix DS-11 spectrophotometer and determined to have *A*_260/280_ and *A*_260/230_ ratios between 1.7 to 2.2 and 1.8 to 2.4. cDNA was synthesized from 1,500 ng of total RNA using a High-Capacity cDNA reverse transcription kit following the manufacturer’s instructions (Applied Biosystems catalog no. 4368813).

### qPCR analysis.

Reverse-transcribed cDNA was diluted 6× and isolated D. melanogaster DNA was diluted 10× in nuclease-free water and used for qPCRs with the Power SYBR green kit (Applied Biosystems, catalog no. 4368702). The following primers were used in this study (Table 1). For analysis of gene expression, *RpLP0* used as the endogenous reference gene because it was identified as the most stably expressed reference gene (29). The *Duox* primers were designed in this study and are exon spanning for *Duox* mRNA (NM_001273039.1). For qPCR analysis of total bacteria and the ratio of *Acetobacter* to *Lactobacillus*, *Dros_rt_1* (*Drosophila* actin gene) was used as the endogenous control. The vehicle (DMSO) group was used as the calibrator in all qPCR analysis experiments, except for the LGR-1 supplementation experiments, where the vehicle groups were used as the calibrators for the respective IMI exposure groups. Reagent volumes for 10-μl reactions (performed in triplicate technical replicates) consisted of 2.5 μl of diluted DNA or cDNA, 5 μl of Power SYBR (2×), and 2.5 μl of forward and reverse primer mix (3.2 μM each stock). Reaction conditions were 50°C for 2 min, then 95°C for 10 min, followed by 40 cycles of 95°C for 15 s and 60°C for 1 min. qPCR was performed on a QuantStudio 5 real-time PCR system (Thermo Fisher Scientific) and analyzed using the associated QuantStudio Design and Analysis software v1.4.3 (Thermo Fisher Scientific). Gene expression (2^−ΔΔ^*^CT^*) was calculated using fold change, and statistical analyses were performed on the −ΔΔ*C_T_* values (50). PCR efficiencies were calculated using LinRegPCR version 2016.1 and determined to be above 1.80. Primer specificity was tested using gel electrophoresis (see Fig. S2A to C in the supplemental material) and monitored by analyzing the melt curves.

**TABLE 1:** qPCR primers used in this study

Primer	Sequence^*a*^	Amplicon size (bp)	Efficiency
*Dros_rt_1* (*Drosophila* actin gene [51])	F: 5′-GGAAACCACGCAAATTCTCAGT-3′	140	1.96
	R: 5′-CGACAACCAGAGCAGCAACTT-3′		
Universal bacterial primer (52)	F: 5′-ACTCCTACGGGAGGCAGCAGT-3′	172	1.85
	R: 5′-ATTACCGCGGCTGCTGGC-3′		
*Acetobacter* spp. (51)	F: 5′-TAGTGGCGGACGGGTGAGTA-3′	134	1.96
	R: 5′-AATCAAACGCAGGCTCCTCC-3′		
*Lactobacillus* spp. (51)	F: 5′-AGGTAACGGCTCACCATGGC-3′	108	1.98
	R: 5′-ATTCCCTACTGCTGCCTCCC-3′		
*RpLP0* (29)	F: 5′-CCGAAAAGTCTGTGCTTTGTTCT-3′	83	1.85
	R: 5′-CGCTGCCTTGTTCTCCCTAA-3′		
*Duox* (this study)	F: 5′-CATGCGCTCCTTCCACAATG-3′	146	1.82
	R: 5′-CACCAAGAAGAAACAGCCGC-3′		
*p38c* (30)	F: 5′-TACCTATCGCGAGATCCGTCT-3′	225	1.84
	R: 5′-ATGTACTTCAGTCCCCGCAGT-3′		
*Mkp3* (20)	F: 5′-GTGACGCTCGCCTACTTGAT-3′	102	1.82
	R: 5′-GAAGTGGAAGTTGGGCGATA-3′		
*Cad99C* (21)	F: 5′-TCTTCGTGAAGCCAGTGGAC-3′		
	R: 5′-ACGATAGCGGGTTACCGTGC-3′	123	1.84

a F, forward; R, reverse.

10.1128/mBio.01395-19.2 FIG S2 Gel electrophoresis verification of qPCR primer specificity. (A) Primers shown: *RpLP0*, *p38c*, *Mkp3*, *Cad99C*, *Acetobacter* spp., *Lactobacillus* spp., and *Dros*_*rt*_*1* (*Drosophila* actin). (B) Primer shown: Universal Bacterial primer. (C) Primer shown: Duox. Download FIG S2, TIF file, 0.9 MB. Copyright © 2019 Chmiel et al. 2019 Chmiel et al. This content is distributed under the terms of the Creative Commons Attribution 4.0 International license.

### LGR-1 IMI tolerance assay.

LGR-1 was grown overnight in MRS and subcultured (1:100) into 96-well plates (Falcon, catalog no. 35177) containing MRS with or without vehicle (DMSO) or 100 ppm IMI. Plates were incubated at 37°C for 24 h and measured every 30 min at 600 nm using a microplate reader (BioTek, Eon).

### Pesticide metabolism/binding assay.

High-performance liquid chromatography (HPLC) analysis of culture supernatant was employed to test if LGR-1 was able to reduce the amount of IMI in culture supernatant. LGR-1 cells grown in minimal medium [2.5 g/liter yeast extract, 1.5 g/liter K_2_HPO_4_, 0.5 g/liter KH_2_PO_4_, 0.5 g/liter (NH_4_)_2_SO_4_, 0.5 g/liter NaCl, 0.4 g/liter MgSO_4_·7H_2_O, 0.05 CaCl_2_, 0.03 g/liter FeSO_4_·7H_2_O] and minimal medium alone were spiked with 100 ppm of IMI and incubated anaerobically for 24 h at 37°C, with shaking (175 rpm) and protected from light. The solutions were then centrifuged at 5,000 rpm (4,500 × *g*) for 10 min at room temperature. Supernatants were removed and filter sterilized using 0.45-μm-pore filters prior to HPLC analysis.

All samples and standards were analyzed using an Agilent 1100 HPLC device equipped with a degasser (G1379A), quaternary pump (G1311A), autosampler (G1313A), and diode array detector (G1315B). All analyses were performed on an Agilent Poroshell 120 EC-C18 (4.6- by 150-mm inside diameter [i.d.], 4-μm particle size) column kept at ambient temperature. The acetonitrile (Fisher catalog no. A996-4) and water (Fisher catalog no. W5-4) used were HPLC grade. The mobile phase consisted of an isocratic mixture of acetonitrile and water (40:80 [vol/vol]) at a flow rate of 1.0 ml/min. The sample injection volume was 5 μl, and detection was performed at 270 nm. Run times were 5 min, with imidacloprid eluting at ∼2.3 min. Data were analyzed using ChemStation A.10.02. The peak area of samples was compared with the peak area of the external calibration curve (1 to 200 ppm) to determine IMI quantification.

### Statistical analyses.

All statistical comparisons were performed using GraphPad Prism 7.0 software. Nonparametric data were statistically compared with an unpaired, two-tailed Mann-Whitney test. Data with unique values were tested for normality using the Shapiro-Wilks test or D’Agostino and Pearson normality test. Normally distributed data were compared with an unpaired, two-tailed *t* test. Experiments with two factors were statistically compared with a two-way analysis of variance (ANOVA), complemented with Sidak’s multiple-comparison test.
