# Supplementary figures and images for: Deleterious Effects of Neonicotinoid Pesticides on Drosophila melanogaster Immune Pathways
Source: mBio. 2019 Oct 1;10(5):e01395-19. doi: 10.1128/mBio.01395-19 (PMC6775452; doi:10.1128/mBio.01395-19)

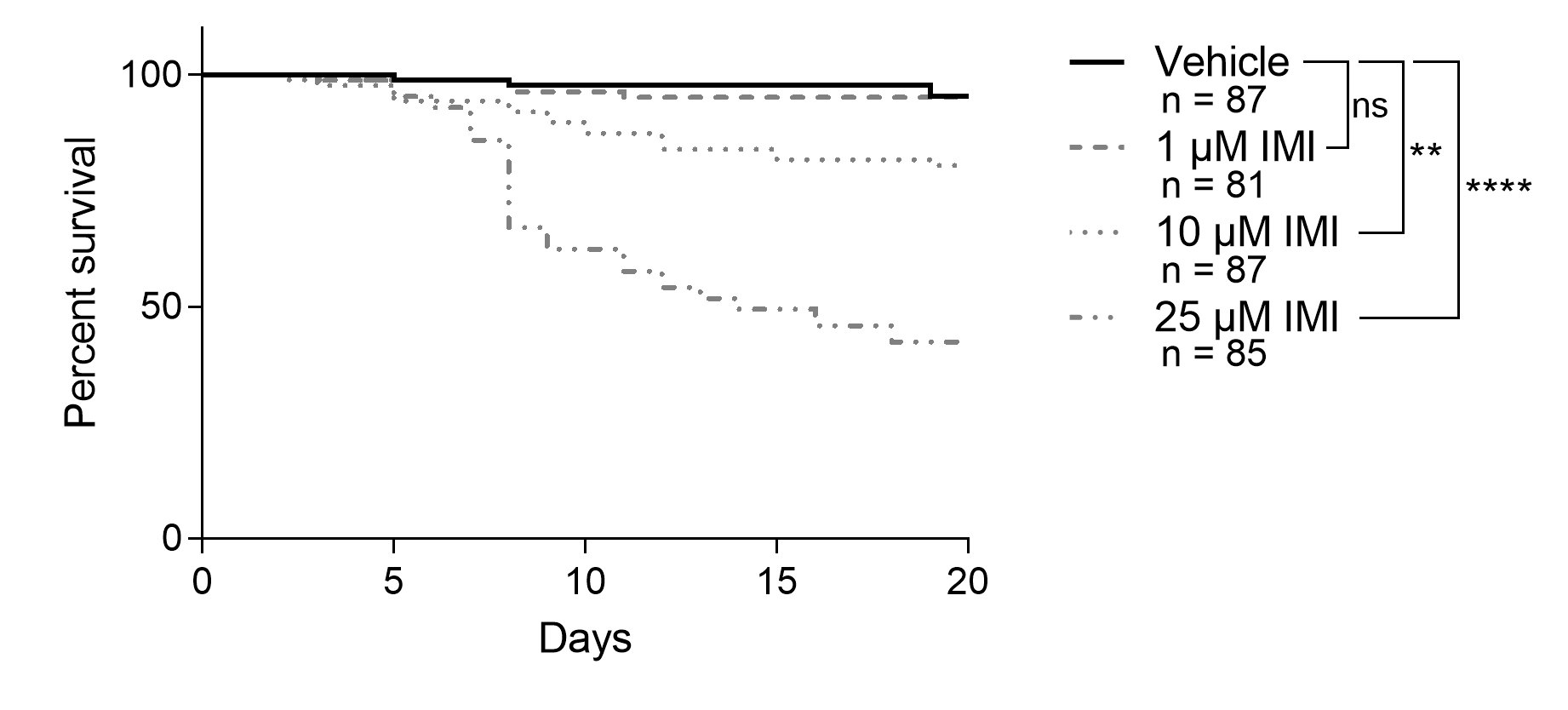

Supplement: FIG S1 [file mBio.01395-19-sf001.tif]

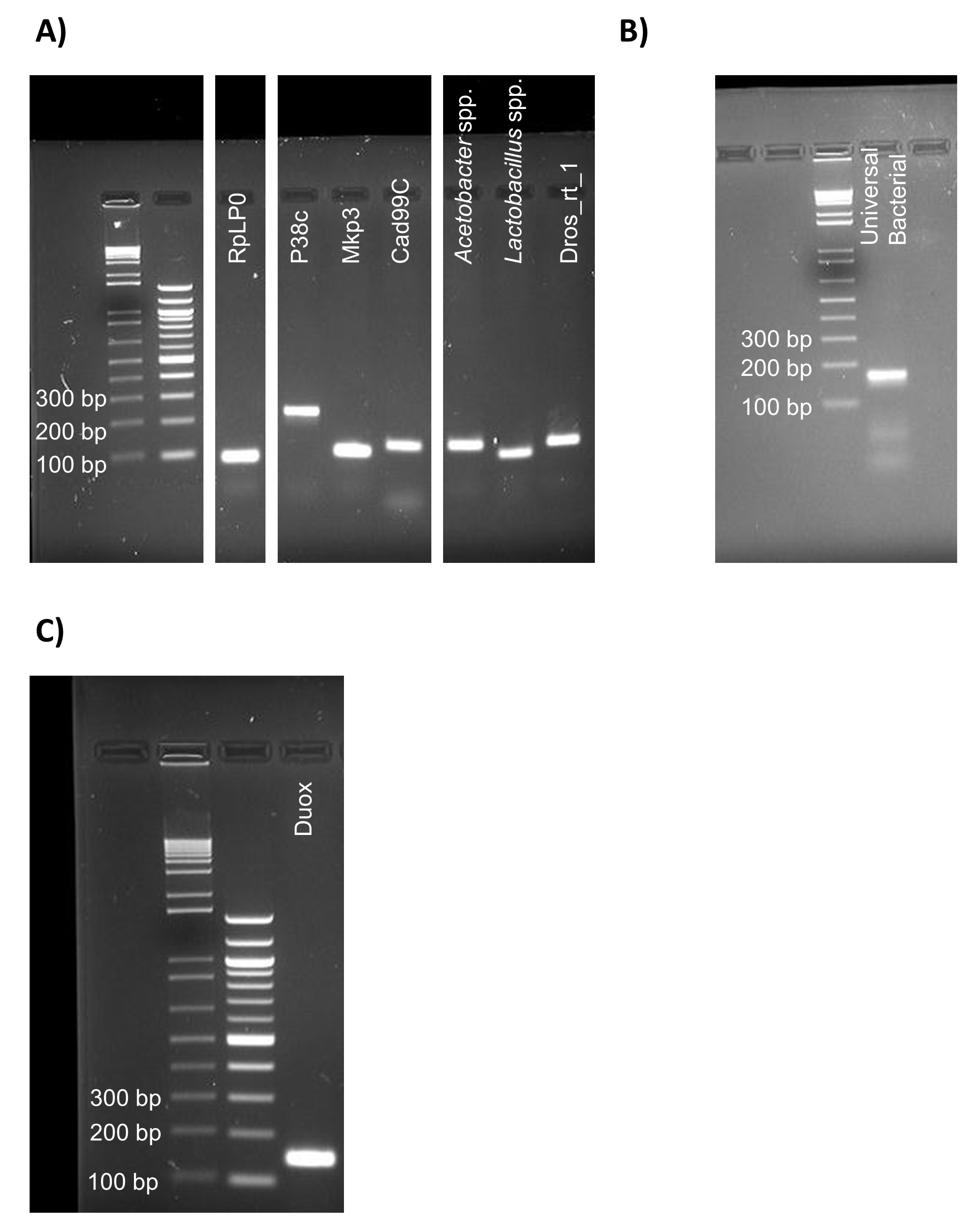

Supplement: FIG S2 [file mBio.01395-19-sf002.tif]
